# The Microbiological Context of HIV Resistance: Vaginal Microbiota and Mucosal Inflammation at the Viral Point of Entry

**DOI:** 10.1155/2012/131243

**Published:** 2012-03-14

**Authors:** John J. Schellenberg, Francis A. Plummer

**Affiliations:** ^1^Department of Medical Microbiology, Faculty of Medicine, University of Manitoba, 260-727 McDermot Avenue, Winnipeg, MB, Canada R3E 3P5; ^2^National Microbiology Laboratory, Canadian Science Centre for Human and Animal Health, 1015 Arlington Street, Winnipeg, MB, Canada R3E 3R2; ^3^Department of Medical Microbiology, University of Nairobi, P.O. Box 30197-00100, Nairobi, Kenya

## Abstract

Immune activation is increasingly recognized as a critical element of HIV infection and pathogenesis, causing expansion of virus founder populations at the mucosal port of entry and eventual exhaustion of cellular immune effectors. HIV susceptibility is well known to be influenced by concurrent sexually transmitted infections; however, the role of commensal vaginal microbiota is poorly characterized. Bacterial vaginosis (BV) is a risk factor for HIV acquisition in studies worldwide; however, the etiology of BV remains enigmatic, and the mechanisms by which BV increases HIV susceptibility are not fully defined. A model of how vaginal microbiota influences HIV transmission is considered in the context of a well-established cohort of HIV-exposed seronegative (HESN) commercial sex workers (CSW) in Nairobi, Kenya, many of whom have increased levels of anti-inflammatory factors in vaginal secretions and reduced peripheral immune activation (immune quiescence). Elucidation of the relationship between complex microbial communities and inflammatory mucosal responses underlying HIV infection should be a priority for future prevention-focussed research.

## 1. Introduction

Fatal opportunistic infections associated with what is now known as the acquired immunodeficiency syndrome (AIDS) were first described in 1981 among young, previously healthy homosexual men in the United States [1]. While global media and health authorities were focussed on stigmatized “risk groups” in North America, largely heterosexually-transmitted epidemics were already well established throughout sub-Saharan Africa by the time the human immunodeficiency virus (HIV) was discovered in 1983. A cohort-based study of commercial sex workers (CSW) in Nairobi, Kenya found that HIV prevalence increased from 4% to over 60% between 1981 and 1985 [2]. Follow-up work identified a subgroup of CSW who were never infected with HIV despite years of exposure, one of the earliest described examples of highly HIV-exposed seronegative (HESN) individuals in the world [3].

 HIV infection results in profound, multifactorial immune dysregulation eventually leading to AIDS usually after a period of 7–10 years. This lengthy asymptomatic period is characteristic of the lentiviridae family of viruses to which HIV and a wide variety of simian immunodeficiency viruses (SIV) belong. Progressive destruction of CD4+ T helper (Th) cell populations via direct viral killing is generally believed to explain slow disease progression; however, recent studies in nonhuman primates have revealed that critical events in the earliest phases of infection determine SIV/HIV immunopathogenesis—a “mucosal catastrophe” [4–7].

The purposes of this review are to describe dynamics of mucosal inflammation as critical determinants of HIV transmission and AIDS pathogenesis, influences of vaginal microbiota as the specific “microbiological context” in which HIV transmission occurs, and how these factors may interact to block HIV transmission in highly HIV-exposed seronegative (HESN) commercial sex workers from Nairobi, Kenya.

## 2. The Nature of HIV Resistance

 Although rarely reported in non-human primates challenged with SIV [8], individuals in diverse groups worldwide appear to resist HIV infection despite repeated exposure. This variability in susceptibility to HIV infection and/or its pathogenic consequences has been intensively studied and found to correlate with a variety of viral, genetic, immunological, and sociobehavioural variables; however, the precise mechanisms of protection in HESN individuals have not been elucidated. Study of this phenomenon is hampered by lack of a clear terminology or definition of who should or should not be classified as resistant to HIV, difficulties in comparing groups of individuals, and the small numbers of individuals included in these studies [9, 10].

 Results from some studies indicate that cell-mediated responses to HIV, possibly subsequent to an aborted infection, may result in protection against the establishment of HIV systemically [11]. Late seroconversion of HESN individuals with preexisting HIV-specific CTL responses, possibly following a break from CSW, indicates that ongoing exposure to CSW and/or HIV may be required to maintain the resistant phenotype [12]. Intriguingly, HIV resistance has also been observed in mothers and sisters of HESN cohort members [11]. Single nucleotide polymorphisms (SNP) in noncoding regions of the interferon regulatory factor- (IRF-) 1 gene in HESN CSW correlate with reduced expression of IRF-1 [13]. Among other effects, reduced IRF-1 production may lead to generalized hyporesponsiveness to proinflammatory stimuli mediated by IFN*γ*. Generally lower levels of basal gene transcription and downregulation of pro-inflammatory cytokines in Th from HESN CSW suggest a “quiescent” immune profile [14]. Reduced activation of Th/CTL and increased frequency of Tregs in HESN compared to other HIV− individuals indicates that increased Treg may potently downregulate Th activation levels [15].

 Screening of cervicovaginal lavage (CVL) protein samples from individuals in the Majengo cohort revealed increased concentrations of the anti-inflammatory serine protease inhibitor trappin-2 (also known as elafin) in CVL from HESN compared to other HIV− and HIV+ individuals [16]. Recently, the epithelial origin and suspected antiviral properties of this molecule were confirmed, with studies showing direct inactivation of HIV particles during coincubation with trappin-2 [17]. Further proteomic studies have identified upregulation of several other antiproteases in vaginal fluid of HESN individuals, including serine protease inhibitors and cystatin, a known anti-HIV factor [18].

 Multiple and often conflicting immunological influences in systemic and mucosal compartments have been investigated to gain insight into the HESN phenotype. Although little consensus has emerged, the “immune quiescence” hypothesis offers a plausible biological explanation for why viral populations at points of entry do not engender productive infections in HESN individuals.

## 3. Mechanisms of Vaginal HIV Transmission

**Figure 1:**
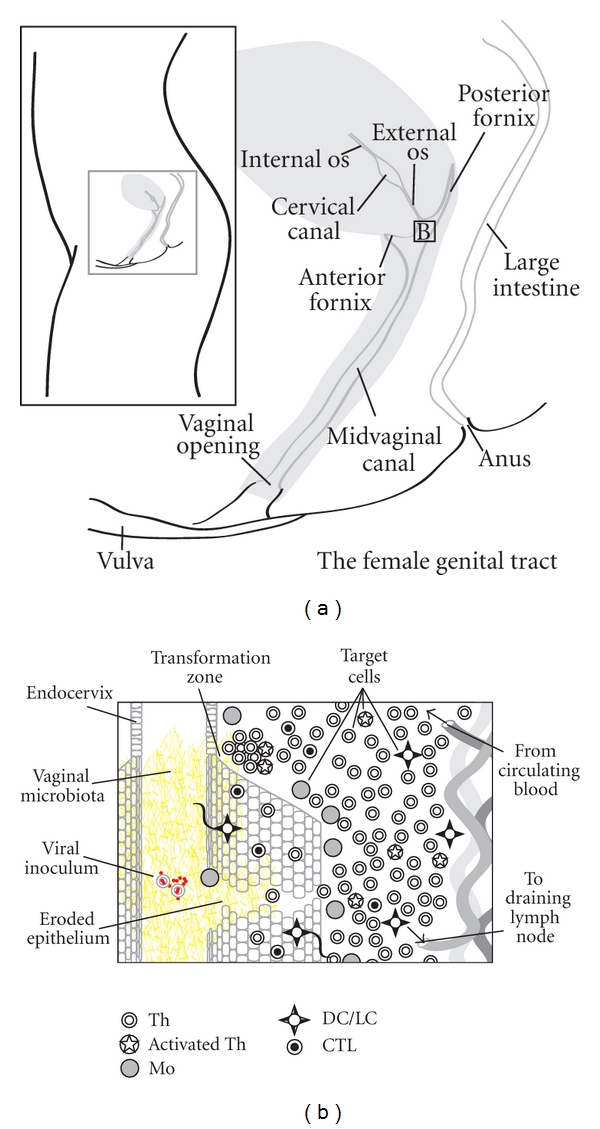
Mechanisms of vaginal HIV transmission. (a) Overview of the major anatomical features of the female genital tract (FGT). (b) Overview of hypothetical mechanisms of HIV transmission in the female genital tract. See text for details.

 The efficiency of male-to-female sexual transmission is very low, in the range of one productive infection for every 200–2000 exposures [19], demonstrating the formidable physicochemical and immunological barrier of the female genital tract (FGT) (Figure 1(a)). Semen from infected individuals, especially those with bacterial coinfections, is known to contain very high concentrations of free virus as well as large numbers of infected lymphocytes and monocytes that may directly transfer infectious virus to vaginal epithelium, T-helper (Th) or antigen-presenting cells such as macrophages (Mo), and Langerhans/dendritic cells (LC/DC) at the mucosal point of entry (Figure 1(b)). The first barrier that the virus must cross is the mucus layer, a dense multilayered network of glycoprotein molecules (mucins) colonized by high concentrations of diverse micro-organisms (vaginal microbiota). As well as creating a dense fluid gel overlying the epithelium, mucins bind covalently to epithelial cells to create a tightly adherent layer called the glycocalyx [20]. Both cell-free and cell-associated virus may become trapped in these networks and subsequently inactivated by low pH and hydrogen peroxide (H_2_O_2_) originating from specific types of bacteria, or by epithelial-derived proteins with antiviral properties, such as defensins or secretory leukocyte protease inhibitor (SLPI) and related effectors [7, 21]. Even under artificial infection conditions with a very high inoculum of pure virus, only a tiny proportion of virions gain access to susceptible cells in the epithelium and LP [7], with productive infection frequently initiated by a single viral particle [22]. Changes in the amount and consistency of excreted mucus, the profile and activity of colonizing microbiota, the amount and nature of epithelial defence molecules due to phase of menstrual cycle, sexual intercourse, coinfections, stress, and nutrition are all likely to influence the likelihood that HIV can cross this initial physical barrier.

 Several mechanisms for crossing the stratified squamous epithelium of the vagina and ectocervix or the simple columnar epithelium of the endocervix and upper reproductive tract to access target cells have been proposed; however, the relative importance of each during infection *in vivo* has not been defined. Both HIV and SIV enter target cells in the FGT primarily after binding the CD4 cell-surface glycoprotein (receptor) and the chemokine ligands CCR5 or CXCR4 (co-receptors) found on target cells throughout FGT epithelial surfaces and lamina propria (LP); however, distribution of various immune cell types has been shown to vary greatly between individuals and anatomical sites, and is strongly influenced by mucosal inflammation [23]. Normal vaginal epithelium is characterized by intraepithelial CD8+ cytotoxic lymphocytes (CTL), relatively few Th restricted to the LP, and low numbers of LC in basal epithelial layers. However, greatly increased intraepithelial Th, and LC are observed in women with chronic vaginal inflammation [23]. The highest concentrations of Mo, Th, and CTL are found associated with the “transformation zone” (TZ) of the exocervical os (Figure 1(b)), where stratified epithelium abruptly ends and the endocervical monolayer begins, indicating a critical position in immune control for ascending pathogens and also increased vulnerability to HIV infection [23]. These observations suggest distinct “immune microenvironments” associated with different anatomical sites within the FGT. Although the cervix is likely to be a preferred site of entry for HIV given higher concentrations of target cells in these regions, especially in the TZ, large increases in numbers of target cells in the vaginal epithelium observed during inflammation increase vulnerability to HIV infection in these tissues as well.

 Penetration of the epithelial layer has been shown to happen within minutes of exposure in experiments with non-human primates [19]. HIV binds lipid moieties such as galactosyl cerebroside (GalC) or other receptors on vaginal epithelial cells [24], leading to transcytosis across or productive infection of the epithelial layer [25]. Intraepithelial and submucosal APC such as LC and DC are thought to play an important role in viral transport to large populations of susceptible cells in the lymphatic tissue. Terminal mannose residues on HIV surface glycoproteins bind to related receptors or lectins such as langerin in intraepithelial LC [26]. Since LC form a tight network at the mucosal surface, and are known to sample the luminal space with specialized cell extensions [19], they are likely to be among the first cells to come into contact with HIV. Viral binding to langerin has been found to trigger virus degradation and block *cis*-infection via an endosomal pathway that is unique to LC. In the absence of activating stimuli, LC resist infection with HIV and transmit virus much less efficiently to Th when compared to submucosal DC. However, activation of LC by TLR stimulation and epithelial production of the cytokine TNF*α* in the context of vaginal coinfections abolishes the protective role of langerin and facilitates *cis*-infection and migration of LC to lymphatic tissue [26].

 Although the precise pathway travelled by HIV during the majority of sexually transmitted infections is unknown, it is clear that genital coinfections or other factors that (1) increase shedding of free virus and infected cells by the infecting partner, (2) increase permeability of the protective mucus layer, (3) cause changes in the composition and function of vaginal microbiota, (4) disrupt the physical integrity of the epithelial barrier, or (5) increase the abundance of target cells at the mucosal port of entry, will increase the likelihood that HIV will be transmitted during heterosexual intercourse.

## 4. Early Tolerogenic Signals and Mucosal Catastrophe: A Shifting Paradigm

**Figure 2:**
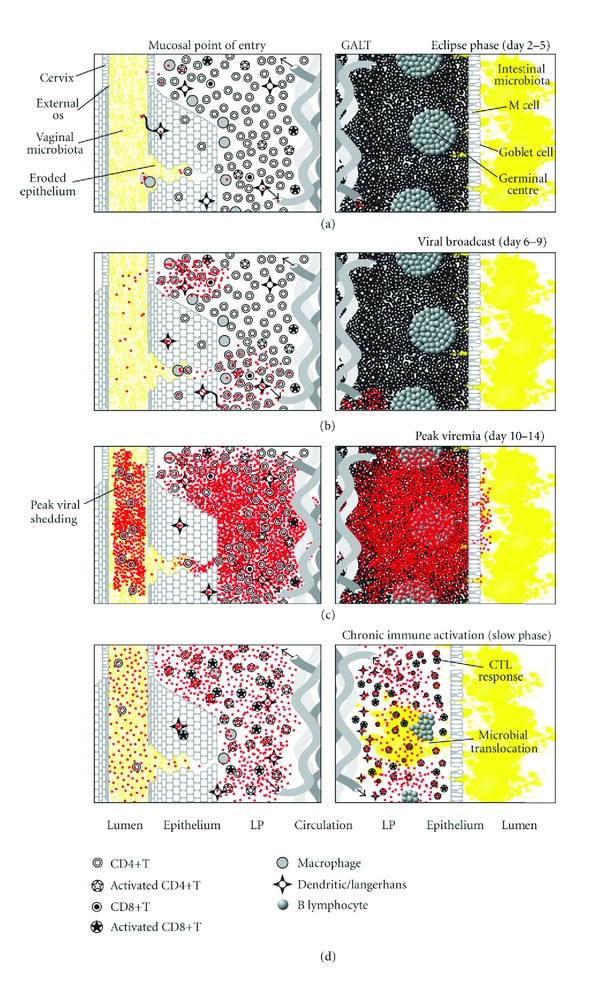
A mucosal catastrophe. Overview of the “fast phase” of HIV infection in the days following infection, including the eclipse phase (a), the viral broadcast phase (b), peak viremia (c), and subsequent chronic immune activation (d). See text for details.

 Once founder populations of virus have adsorbed and penetrated target cells at the port of entry, there follows a period of several days in which there is little or no productive expansion of viral populations. This “eclipse phase” (Figure 2(a)) represents a critical opportunity to mitigate expansion of very small populations of virus in spatially dispersed foci amid a relatively small concentration of target cells in lamina propria (LP) relative to lymphoid tissue [7]. Inflammation and immune activation near the port of entry are likely to determine expansion or decay of HIV founder populations [27]. If the virus reproductive rate exceeds unity (i.e. each infected cell results in the infection of at least one other cell), the threshold to productive infection (the “fast phase”) is crossed on or about day seven (Figure 2(b)). The virus-as-signal is amplified and broadcast distally from the port of entry in LP to draining lymph nodes, the systemic circulation, and all lymphatic tissue, rising to peak levels by day ten to fourteen [7] (Figure 2(c)). As a result of direct viral killing and Fas/FasL-mediated apoptosis in the presence of high concentrations of viral gp120, mucosal Th populations, concentrated in gut-associated lymphoid tissue (GALT), vanish almost entirely within four days [5, 7]. This massive immunological insult is not reflected in the systemic circulation, where only a minority of Th reside, explaining earlier models of gradual Th depletion in disease progression based on measuring Th in peripheral blood only [7].

 The “slow phase” of infection (Figure 2(d)) following the initial insult is marked by chronic, generalized immune activation, including increased activation and turnover of Th, and increased systemic proinflammatory cytokines [28]. Immune activation has been found to be a better predictor of disease progression than viral load, but whether the virus drives immune activation or vice versa is unresolved. Immune activation increases target cells for HIV infection and the self-perpetuating relationship between activation and viral replication leads to eventual clonal exhaustion of Th memory pools, as reflected in rising viral loads and declining Th counts as disease progresses [28]. Although antiretroviral therapy can mitigate immune defects by reducing viral replication, continued immune activation remains a barrier to immune reconstitution following treatment [28]. In “elite controllers,” defined as HIV-infected individuals who maintain very low viral loads in the absence of treatment, progressive loss of Th correlates with immune activation [28]. Therefore, regardless of virus control in later stages of infection, chronic immune activation continues to strongly influence disease progression, likely due to translocation of immune-stimulating microbial products such as lipopolysaccharide (LPS) across damaged mucosal barriers in the gut. Consistent with this hypothesis, a recent study has found higher LPS levels in the blood of chronically HIV-infected people [29].

 In contrast, many SIV, including SIVagm from African green monkeys, cause no immunopathology in their natural hosts, despite intense viral replication and massive depletion of Th in systemic and mucosal compartments during acute infection [30, 31]. Surprisingly, this initial insult to Th populations does not result in enteropathy or chronic immune activation in nonpathogenic infections, and the function of various immune cell subsets is preserved. Although Th numbers in peripheral blood and mucosal tissues rise in the weeks following acute infection by both pathogenic and nonpathogenic SIV, steep declines in the chronic phase are only observed in pathogenic SIV infection. No increases in Th activation or apoptosis are observed in chronic nonpathogenic SIV infection, while activation and proliferation of CTL occurs early and then subsides, with very little detectable LPS in serum indicating preservation of the gut barrier and absence of microbial translocation. Upregulation of inflammatory cytokines and beta chemokines is not observed in serum of SIVagm-infected green monkeys [31, 32]. Instead, upregulation of TGF-*β* stimulates expression of *FoxP3* in Th and CTL within one day of infection, leading to high levels of immunosuppressive IL-10 at early timepoints [32]. This early induction of the T regulatory (Treg) subset and IL-10 may counteract immune activation signals due to chronic infection in SIVagm-infected green monkeys.

 An altered balance between Treg populations and the related Th17 subset has also been identified in pathogenic SIVmac infection of the pigtailed macaque, but not in nonpathogenic SIVagm infection of the green monkey [33]. Th17 cells express IL-17 and IL-22, cytokines that have been shown to be important for immune responses to extracellular bacteria in the gut, where Th17 cells are concentrated [34]. IL-17 induces IL-8 to promote recruitment of neutrophils to sites of bacterial infection, while IL-22 induces proliferation of intestinal epithelial cells and repair of the mucosal barrier, as well as stimulating epithelial production of antibacterial defensins. Th17 cells and Treg are derived from a common progenitor cell and their reciprocal differentiation depends on the cytokine milieu generated by DC with TLR stimulated by bacterial products in peripheral tissues [33]. In humans, the Th17 phenotype is induced in naïve T cells in the presence of IL1*β* and IL-6 [35]. Interestingly, most *α*4*β*7+ cells in GALT of rhesus macaques have been shown to be Th17 cells [36]. Therefore, preferential depletion of Th17 cells in pathogenic SIV, as well as in HIV infection [34], may be due to the affinity of SIV/HIV gp120 for *α*4*β*7 integrin on gut mucosal Th, leading to permeability of mucosal barriers in the gut, microbial translocation, and chronic immune activation. The early upregulation of Treg in nonpathogenic SIV infection may contribute to balanced ratios of Th17/Treg cell populations in GALT and mitigate immune dysfunction leading to AIDS [34].

 These observations strongly suggest that the timing and pattern of host immune responses to infection play a critical role in determining disease outcome. Induction of early tolerogenic responses to founder populations may block virus propagation and broadcasting into systemic compartments, or mitigate damage to mucosal Th populations thereby reducing immune activation resulting from microbial translocation. Immune activation at compromised mucosal surfaces is a critical component of both vulnerability to vaginal HIV transmission and AIDS pathogenesis in the gut; however, nothing is known about the dynamics of normal microbiota in these contexts.

## 5. The Microbiological Context: A Model

**Figure 3:**
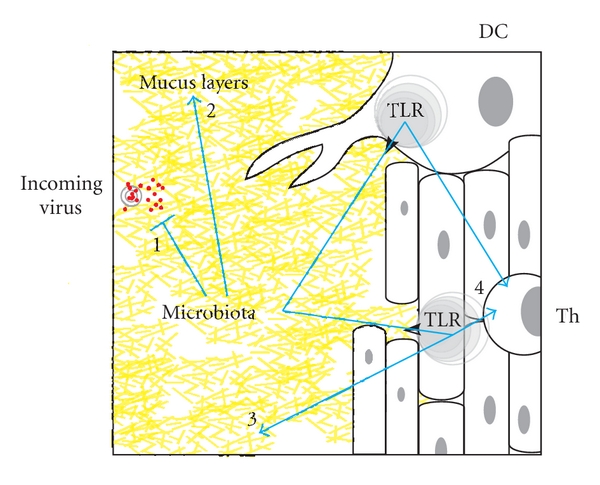
The microbiological context of HIV resistance. Vaginal microbiology influences susceptibility of the mucosal layer to HIV infection in multiple interrelated ways, including (1) direct effects on viral particles or infected allogenic cells via binding, mannose scavenging, or bacterial metabolites (acid, H_2_O_2_), (2) alteration of quantity/viscosity/permeability of mucin network in mucus layer via proteases, (3) stimulation of innate mucosal defence molecules and cells via TLR stimulation, and (4) modulation of T-cell activation and susceptibility via conditioning by TLR-stimulated dendritic cells, macrophages, or epithelial cells.

 Vaginal microbiology is predicted to influence susceptibility of mucosal surfaces to HIV infection via a number of direct and indirect mechanisms (Figure 3). Genital tract infections such as chlamydia, gonorrhea, syphilis, herpes simplex virus (HSV), human papilloma virus (HPV), and trichomoniasis are known to increase the risk of HIV acquisition [37–40]. The “enigmatic” clinical entity known as bacterial vaginosis (BV) [41, 42], associated with increased susceptibility to many sexually transmitted infections, has been shown to increase risk for HIV infection in numerous studies from around the world [43]. BV is a frequently asymptomatic clinical condition defined by a reduction in vaginal *Lactobacillus* populations and overgrowth of anaerobic and Gram-negative organisms [42]. Increasingly linked with negative reproductive health outcomes, such as miscarriage, premature birth, postoperative infections, pelvic inflammatory disease, and HIV infection, its etiology and clinical course remain poorly defined with a consequent lack of effective prevention and treatment strategies [41, 44, 45].

 The current “gold standard” for diagnosis of BV is based on observation of clinical markers, known as Amsel's criteria, including thin, milky discharge, high vaginal pH, presence of “clue cells” (shed epithelial cells covered in Gram-negative bacteria), and amine (“fishy”) odour [46]. In the laboratory setting, most studies rely on scoring profile of microbial types in Gram-stained vaginal swab smears [47]. These systems are based on the relative abundance of *Lactobacillus* morphotypes (short to long, straight-sided rods) compared to BV bacteria morphotypes (Gram-negative/variable coccobacilli and curved rods).

 Quantitative bacteriology and microbial ecology are of particular concern in understanding BV, which is defined by changes in levels of total bacteria and alterations in abundance of bacterial subpopulations, rather than the presence of a specific pathogen [44]. For example, BV-associated bacteria such as *Gardnerella*, *Mobiluncus*, *Atopobium,* and *Mycoplasma* species are routinely isolated from women without BV [45]. Simultaneous isolation of *Mycoplasma*, *Gardnerella,* and anaerobes, described as a “pathologic core,” combined with failure to isolate *Lactobacillus* in a vaginal sample, has been found to be strongly associated with BV [48]. Despite a general lack of external signs of inflammation in BV (as indicated by the -*osis* suffix signifying overgrowth, rather than the -*itis* suffix signifying inflammation) [49], many studies associate BV with increased proinflammatory signalling via TLR stimulation [50–52]. BV is characterized by an increase in the overall bacterial load in the vagina, with possible implications for TLR stimulation and immune activation. The presence of various proteolytic enzymes during BV demonstrate the importance of mucin lysis as a source of energy for the bacterial consortium [53], and may also explain nutritional synergies observed in culture-based experiments with BV bacteria [54]. Enzymes such as sialidase and prolidase have been shown to impair innate and adaptive immune effectors in the vagina, including cytokines, immunoglobulins, and cellular receptors, and are likely to explain lack of overt signs of inflammation despite local production of pro-inflammatory signals during BV [55]. Thinning of the vaginal mucus layer may also increase opportunities for HIV to access target cells in underlying mucosa.

 Given a preeminent ecological position in the vaginal environment [56], colonization of the female genital tract by *Lactobacillus* species is increasingly recognized as critical for overall vaginal health and resistance to infection by bacterial and viral pathogens, including HIV [57]. Under the influence of estrogen, vaginal epithelial cells store glycogen, which is hydrolysed to glucose and metabolized by vaginal bacteria [58]. Excreted lactate, especially by *Lactobacillus* species, reduces the overall pH of the vaginal lumen to “normal” levels, in the range of 3.9 to 4.5 [59, 60]. Along with effectors such as H_2_O_2_ and bacteriocins, acid production by *Lactobacillus* is believed to discourage overgrowth of other bacterial genera including *Streptococcus*, *Gardnerella*, *Bacteroides,* and *Mycoplasma* [61]. H_2_O_2_ produced by a *Lactobacillus* strain has been shown to inactivate HIV *in vitro* [62]. Production of acid and H_2_O_2_ may be synergistic, since bacterial H_2_O_2_ is more likely to remain stable at lower pH *in vitro *[63]. The ability of *Lactobacillus* to produce acid from mannose scavenged as a carbon source has also been hypothesized to block HIV infection through digestion of mannosylated residues on viral glycoproteins [64]. Interestingly, two promising molecules currently being studied as potential topical agents for blocking HIV infection (microbicides) also bind mannose residues on the viral surface [65, 66].

 Absence of culturable H_2_O_2_-producing *Lactobacillus* has been shown to be an important contributing factor in elevated risk for cervicitis observed in those with BV [67]. This finding confirms that BV-related organisms are associated with an elevated risk of genital tract inflammation, the converse of which may be a non/anti-inflammatory role for *Lactobacillus* organisms. The possibility of anti-inflammatory effects by *Lactobacillus* organisms in the vagina is all but unexplored, although several studies concerning the gut epithelium indicate induction of epithelial defence molecules, anti-inflammatory signalling, and increased Treg by specific strains of *Lactobacillus* [55, 68–70].

 We recently conducted the first study to address BV and *Lactobacillus* colonization specifically as possible factors contributing to HIV resistance in highly exposed commercial sex workers fron Nairobi [71]. Compared to other HIV− individuals, HESN in the Majengo cohort were just as likely to be diagnosed with BV and had similar vaginal pH in a cross-sectional study, a finding confirmed by retrospective analysis of BV diagnoses over several years in nearly 1,000 individuals [71]. Ultradeep molecular characterization of vaginal microbiota also revealed few differences in species distribution between HESN and other HIV− individuals [72]; however, neither study was conclusive. BV is known to induce a pro-inflammatory environment and create vulnerability to HIV, so the ability of an individual to modulate some aspect of the inflammatory process related to the BV cycle may determine her ability to block viral infection. Longitudinal studies examining dynamics of vaginal microbiota and mucosal responses over time will be necessary in order to definitively address the potential impact of vaginal microbiology on HIV resistance in the Majengo cohort.

## 6. Conclusions

 The immune activation hypothesis of HIV pathogenesis suggests that multiple factors may contribute to reduced viral broadcast at the mucosal port of entry, either blocking productive infection entirely or limiting damage to lymphoid tissue subsequent to peak viremia. Therefore, these phenomena may be seen as being part of a continuum of HIV resistance [10]. HESN CSW in the Majengo cohort are among the most striking and well-studied examples of HIV resistance worldwide. Ongoing characterization of this group has revealed many immunogenetic factors that may contribute to relative resistance in a subgroup of CSW, including recent findings indicating a role for reduced T-cell activation or “immune quiescence.” Mucosal studies have revealed the possibility that HESN CSW may have increased anti-inflammatory factors in mucosal fluids, mirroring reduced immune activation in peripheral blood. Together, these studies suggest a unified set of factors in HESN women that reduce susceptibility of target cells *in vivo* or hamper viral expansion from the mucosal port of entry.

 Although the vaginal microbiota has so far not been found to play a critical role in HIV resistance, the purpose of this review has been to clarify the role that mucosal inflammation and microbiological dynamics play in the earliest stages of HIV disease. In natural infections, the best predictor of immunodeficiency and disease is not lentiviral replication within the host but T-cell activation and activation-induced cell death leading to depletion of critical cell populations. Early immunosuppressive signals and upregulation of T-regulatory cells within 24 hours of infection are characteristic of nonpathogenic infections, despite subsequent intense viral replication and initial depletion of CD4 in blood and lymphoid tissue in both pathogenic and nonpathogenic infections, while CSW who resist HIV infection entirely for long periods appear to have reduced levels of T cell activation and increased anti-inflammatory molecules in vaginal secretions.

 These findings indicate that modulating immune activation, rather than directly interfering with virus, may be an alternative strategy for stopping HIV infection or mitigating pathogenesis. Whether the purposeful application of live microorganisms (probiotics) might be beneficial for these purposes has only begun to explored [73], but similar principles guide the development of HIV microbicides, defined as topical agents applied vaginally to block HIV transmission [74]. Few studies have comprehensively assessed the impact of microbicides on vaginal microbiota, examining only very limited culture-based parameters. In-depth analysis of the microbiological context is likely to be critical for assessment of candidate microbicides on composition, structure, and function of vaginal microbial communities. A concerted effort to generate detailed microbiological profiles and link this information to mucosal immune parameters will inform upcoming clinical trials for “second generation” microbicides, including probiotics, as potentially effective tools for HIV prevention.

Central questions of HIV prevention-related research in coming years might be how do microbiological and host factors interact with each other as part of the normal functioning of mucosal surfaces exposed to a wide variety of endogenous and exogenous stimuli? How do these interactions influence the likelihood that HIV introduced at the interface of host and microbiota will cross formidable physicochemical barriers and irreversibly alter host immune function? Many challenges remain to adequately define vaginal microbial community dynamics across individuals and over time.
